# Supplementary material for: Relationship between depression, prefrontal creatine and grey matter volume
Source: J Psychopharmacol. 2021 Oct 26;35(12):1464–72. doi: 10.1177/02698811211050550 (PMC8652356; doi:10.1177/02698811211050550)
Supplement: Supplementary_Materials – Supplemental material for Relationship between depression, prefrontal creatine and grey matter volume [file Supplementary_Materials.docx]

**Supplementary Materials - Relationship Between Depression, Prefrontal Creatine and Gray Matter Volume**

**Methods**

***^1^H-MRS Data Acquisition, Pre-processing and Analysis***

Spectra were analysed using LCModel 6.3-1L, with a basis set consisting of 19 simulated spectra; alanine (Ala), ascorbate (Asc), aspartate (Asp), creatine (Cr), γ-aminobutyric acid (GABA), glucose (Glc), glutamine (Gln), glutamate (Glu), glycine (Gly), glutathione (GHS), glycerophosphocholine (GPC), phosphocholine (PCh), lactate (Lac), myo-inositol (mI), N-acetylaspartate (NAA), N-acetylaspartateglutamate (NAAG), phosphorylethanolamine (PE), scyllo-inositol (Scyllo) and taurine (Tau). We have presented data pertaining to creatine in the main manuscript, and will present data pertaining to glutamate, total choline, myo-inositol and *N*-acetylaspartate below; we will not present data pertaining to the remaining metabolites as such data may be unreliable (i.e. due to there being very low concentrations of these metabolites or a poor model fit). Cramer-Rao Lower Bounds (CRLBs) for each of these 4 metabolites can be seen in table S1.

Metabolites concentrations were corrected using the formula (creatine*(43300*gray matter volume + 35880*white matter volume + 55556*cerebrospinal fluid volume))/(35880*(1 - cerebrospinal fluid volume)); creatine concentrations corrected in this manner are denoted as ‘Creatine *Corr*’. Concentrations of both glutamate and GABA, and the remaining 15 metabolites (for completeness) were also corrected using the same formula.

***sMRI Data Pre-processing and Analysis***

The data were skull-stripped using the standard adaptive probability region-growing approach, normalized to the standard tissue probability map and segmented into gray matter, white matter and cerebral spinal fluid. These images were ‘modulated normalized’ images (i.e. voxel values were modulated using the Jacobian determinant), derived from the spatial normalization so that the absolute volume of gray matter could be examined. This type of modulation requires group analyses to correct for individual differences in brain size; total intracranial volume was therefore added as a covariate to all group-level general linear models. The gray matter tissue segments then underwent statistical quality control testing for inter-subject homogeneity and overall image quality as included in the CAT12 toolbox, before a manual visual inspection procedure for potentially newly-introduced artefacts. These images were then registered to the MNI template using DARTEL registration and smoothed using an 8mm Gaussian Kernel.

***Bayesian Analyses***

For the Bayesian analyses, on the basis of Jeffreys (1961), we considered Bayes Factors (*BF*_10_; NB: not logarithmically transformed) smaller than 1/100 to be extreme evidence for the null hypothesis, a *BF*_10_ between 1/100 and 1/30 to be very strong evidence for the null, a *BF*_10_ between 1/30 and 1/10 to be strong evidence for the null, a *BF*_10_ between 1/10 and 1/3 to be moderate evidence for the null, and a *BF*_10_ between 1/3 and 1 to be not worth more than a bare mention. Conversely, we considered *BF*_10_ larger than 100 to be extreme evidence for the experimental hypothesis, a *BF*_10_ between 100 and 30 to be very strong evidence for the experimental hypothesis, a *BF*_10_ between 30 and 10 to be strong evidence for the experimental hypothesis, a *BF*_10_ between 10 and 3 to be moderate evidence for the experimental hypothesis, and a *BF*_10_ between 3 and 1 to be not worth more than a bare mention.

**Results**

***Influence of Age, Gender, Cannabis and Tobacco Use on Prefrontal Metabolites***

An ANOVA revealed that prefrontal metabolites were not influenced by age (all *p*s > 0.302, all *BF_10_* < 0.543), gender (all *p*s > 0.177, all *BF_10_* < 0.378) or cannabis use (all *p*s > 0.121, all *BF_10_* < 0.822). There was however an expected effect of cigarette smoking on concentrations of prefrontal creatine (*F*(1,75) = 16.641, *p* < 0.001, *BF_10_* = 29.122), prefrontal glutamate (*F*(1,75) = 4.725, *p* = 0.012, *BF_10_* = 4.121), *N*-acetylaspartate (*F*(1,75) = 11.165, *p* < 0.001, *BF_10_* = 18.463) and myo-inositol (*F*(1,75) = 15.205, *p* < 0.001, *BF_10_* = 25.285). Specifically, compared to non-smokers, smokers exhibited lower concentrations of these four metabolites in this brain region. There was no significant effect of cigarette smoking on the remaining metabolites (all *p*s > 0.436, all *BF_10_* < 0.727).

***Depression***

ANOVAs that controlled for age, gender, and daily cannabis and tobacco use revealed no significant correlations between DASS depression scores and concentrations of glutamate (*F*(1,76) = 1.515, *p* = 0.222), total choline values (*F*(1,76) = 1.200, *p* = 0.277), myo-inositol (*F*(1,76) = 1.944, *p* = 0.190) or *N*-acetylaspartate (*F*(1,76) = 2.589, *p* = 0.112).


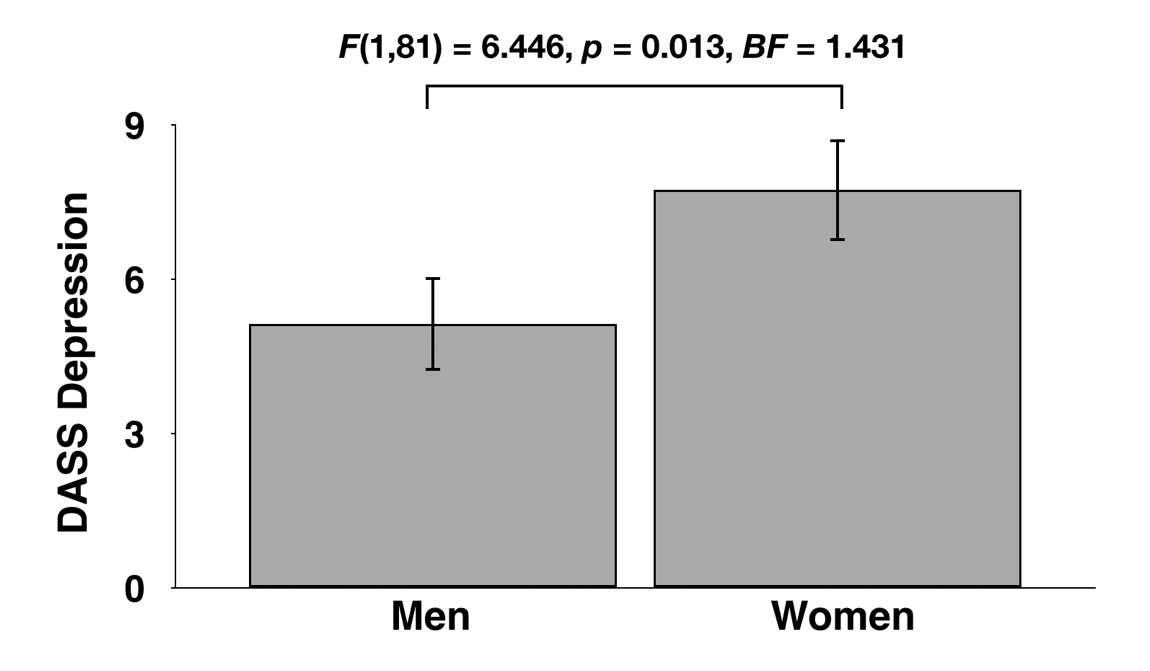


**Fig S1. Depression scores differ between men and women**

***Anxiety***

The mean self-reported score on the anxiety subscale of the DASS was 5.52 (SD = 5.42). Specifically, 57 participants scored between 0-7 and so were classified as ‘non-anxious’, while the remaining 27 participants scored 8+ and were thus classified as ‘anxious; of these, 12 scored between 8-9 and were defined as ‘mildly anxious’, 9 scored between 10-14 and were defined as ‘moderately anxious’, and 6 scored between 15-19 and were defined as ‘severely anxious’. The skewness and kurtosis values for anxiety scores were within acceptable ranges.

An ANOVA to determine main effects of age, gender, cannabis and tobacco use on anxiety scores revealed that magnitude of anxiety was influenced by age (*F*(1,77) = 4.333, *p* = 0.041, *BF_10_* = 1.198), but was not influenced by gender (*F*(1,77) = 0.483, *p* = 0.489, *BF_10_* = 0.619), cannabis use (*F*(1,77) = 0.010, *p* = 0.922, *BF_10_* = 0.148) or tobacco use (*F*(1,77) = 1.221, *p* = 0.301, *BF_10_* = 0.742).

ANOVAs that controlled for age, gender, and daily cannabis and tobacco use revealed no significant correlations between DASS anxiety scores and concentrations of glutamate (*F*(1,76) = 0.541, *p* = 0.464), total choline values (*F*(1,76) = 0.001, *p* = 0.972), myo-inositol (*F*(1,76) = 0.378, *p* = 0.541) or *N*-acetylaspartate (*F*(1,76) = 0.088, *p* = 0.768).

***Stress***

The mean self-reported score on the stress subscale of the DASS was 9.26 (SD = 7.79). Specifically, 69 participants scored between 0-14 and so were classified as ‘non-stressed’, while the remaining 15 participants scored 15+ and were thus classified as ‘stressed’; of these, 5 scored between 15-18 and were defined as ‘mildly stressed’, 4 scored between 19-25 and were defined as ‘moderately stressed’, and 5 scored between 26-33 and were defined as ‘severely stressed’. The skewness and kurtosis values for anxiety scores were within acceptable ranges.

An ANOVA to determine main effects of age, gender, cannabis and tobacco use on stress scores revealed that magnitude of anxiety was not influenced by age (*F*(1,77) = 0.274, *p* = 0.602, *BF_10_* = 0.411), gender (*F*(1,77) = 2.223, *p* = 0.140, *BF_10_* = 0.912), cannabis use (*F*(1,77) = 0.229, *p* = 0.633, *BF_10_* = 0.277) or tobacco use (*F*(1,77) = 0.873, *p* = 0.422, *BF_10_* = 0.610).

ANOVAs that controlled for age, gender, and daily cannabis and tobacco use revealed no significant correlations between DASS stress scores and concentrations of glutamate (*F*(1,76) = 0.214, *p* = 0.645), GABA (*F*(1,76) = 0.181, *p* = 0.671), total choline values (*F*(1,76) = 0.022, *p* = 0.882), myo-inositol (*F*(1,76) = 0.047, *p* = 0.828) or *N*-acetylaspartate (*F*(1,76) = 0.160, *p* = 0.691).

Finally, DASS depression scores were not correlated with Cramer-Rao lower bounds for creatine (*r* = 0.182, *p* = 0.231), glutamate (*r* = -0.042, *p* = 0.705), line width (in Hz) (*r* = -0.130, *p* = 0.237) or signal-to-noise ratio (*r* = -0.126, *p* = 0.254). DASS anxiety and stress scores were not related to any of these variables either (all *p*s > 0.212).

|  | **All Participants** | **Depressed (DASS > 9)** | **Non-depressed (DASS < 10)** |
| --- | --- | --- | --- |
| *N* | 84 | 19 | 65 |
| Cramer-Rao-Bound (Glutamate) | 4.32 (0.74) | 4.56 (0.78) | 4.25 (0.71) |
| Cramer-Rao-Bound (Total Choline) | 3.71 (1.29) | 4.22 (1.66) | 3.58 (1.17) |
| Cramer-Rao-Bound (myo-inositol) | 3.01 (1.20) | 3.39 (2.15) | 2.90 (0.77) |
| Cramer-Rao-Bound (*N*-acetylaspartate) | 3.22 (1.37) | 3.72 (1.27) | 3.05 (1.36) |

**Table S1. Cramer-Rao Lower Bounds for glutamate, total choline, myo-inositol and *N*-aacetylaspartate**
